# Construction of an efficient electroporation transformation system promotes the application of Targetron in wild-type *Paenibacillus elgii* 219

**DOI:** 10.1128/aem.02041-24

**Published:** 2025-04-22

**Authors:** Guangxin Yang, Siyu Li, Yonghang Ma, Siyi Peng, Xiangfang Zeng, Jinxiu Huang, AiHua Deng, Shiyan Qiao, Haitao Yu

**Affiliations:** 1 State Key Laboratory of Animal Nutrition and Feeding, Ministry of Agriculture and Rural Affairs Feed Industry Centre, China Agricultural University 34752 , Beijing, China; 2 Frontier Technology Research Institute of China Agricultural University in Shenzhen, Shenzhen, China; 3 ChongQing Academy of Animal Sciences, Rongchang 580592 , Chongqing, China; 4 National Center of Technology Innovation for Pigs, Rongchang, Chongqing, China; University of Illinois Urbana-Champaign, Urbana, Illinois, USA

**Keywords:** *Paenibacillus*, transformation efficiency, Targetron, glycosyltransferase

## Abstract

*Paenibacillus* species have attracted much attention as a promising cell factory that can yield a variety of bioactive peptides and functional exopolysaccharides. Improving the production capacity of *Paenibacillus* through genetic engineering is crucial for the development of bioactive natural products. This study systematically evaluated the effects of electroporation transformation parameters on the introduction of DNA into *Paenibacillus elgii* 219, which was isolated from soil and preserved in our lab. The transformation efficiency was improved to 1.25 × 10^6^ transformants/μg DNA by optimizing factors including the growth and recovery media content, the growth stages of *P. elgii* 219, electric field strength, electroporation buffer composition, cell wall weakening, and DNA quality. The results represent the highest transformation efficiency reported thus far for *Paenibacillus*. This work further established a group II intron-based gene editing system suitable for *P. elgii* 219 for the first time. As expected, plasmid pDX4383 was successfully transferred into *P. elgii* 219, and a putative glycosyltransferase gene was deleted, thereby eliminating flocculent precipitation during fermentation. Taken together, this study demonstrates that the electroporation-mediated transformation of exogenous DNA combined with group II intron-based gene editing provides an effective approach for the genetic engineering of *Paenibacillus* bacteria.

**IMPORTANCE:**

The establishment of an efficient plasmid DNA transformation protocol for *P. elgii* 219 has established a robust foundation for further in-depth investigations into its physiological and metabolic processes. Additionally, the successful advancement of group II intron-mediated gene editing technology imbues *P. elgii* 219 with the potential to serve as a highly efficient cell factory. These accomplishments furnish novel perspectives and references for the exploration of other *Paenibacillus* species in environmental contexts and the development of their products with economic value.

## INTRODUCTION

Bacterial species belonging to the *Paenibacillus* genus have been studied for decades, and increasing evidence indicates that *Paenibacillus* species represent a potential gold mine of bioactive substance candidates (1, 2). These well-known bioactive compounds, particularly bacterial lipopeptides, are widely applied in agriculture, food, chemical, and biomedical fields. *Paenibacillus elgii* 219 (CGMCC No. 32461), a soil microorganism preserved in our laboratory, plays a vital role in the production of numerous well-known compounds, including the lanthipeptide-class peptide penisin and the cyclic lipopeptide octapeptin. These antimicrobials exhibit remarkable biological activity, with a special emphasis on their potential to combat clinically drug-resistant bacterial infections (3, 4). This unique attribute underscores the immense potential of *Paenibacillus elgii* 219 as a cell factory. Unfortunately, the uncharacterized viscous exopolysaccharides (EPS) yielded by *P. elgii* 219 result in the adhesion of bacterial cells to the culture vessel, limiting both bacterial growth and productivity improvements. To address this issue, an in-depth analysis of EPS biosynthesis in *P. elgii* 219 is necessary. Although the general biosynthetic pathway of exopolysaccharides is well characterized, the specific functions of individual glycosyltransferases involved in the stepwise assembly of the repeating unit remain elusive (5). Analyzing the functions of individual glycosyltransferases based on gene knockout is an effective strategy for gaining detailed insights into the biosynthetic pathway of EPS (6). Therefore, establishing efficient gene editing tools and DNA transformation methods for the host bacterium is of great importance.

Electroporation is generally recognized as the most efficient and convenient approach for the introduction of exogenous DNA into host cells because of its high reproducibility and applicability to multiple species. More importantly, electroporation exhibits a high capacity to transform strains that are difficult or even previously considered impossible to transform, and it holds fundamental significance in bacterial genetic engineering (7, 8). For example, previous studies have shown that *Bacillus* species can efficiently integrate and stably inherit exogenous DNA into their cells when electroporation is utilized (9, 10). However, when applied to wild-type strains isolated from the environment, conversion often proves challenging, especially when plasmids are used to express different proteins and the number of bases increases to complicate the process (11, 12). Thus, the systematic evaluation and optimization of transformation protocols are typically required for specific wild-type strains to achieve the efficient introduction of exogenous DNA. The efficiency of exogenous DNA transformation can be effectively improved by optimizing the growth media, bacterial growth stages, strength of the applied electric field, electroporation buffers, and recovery media, as well as by employing cell wall-weakening agents (7, 11, 13–15).

In recent years, the extensive application of gene editing tools in bacterial genetic engineering has improved the production capacity, with the potential for industrial-scale production (16). Currently, reports on the *Paenibacillus* gene-editing system are limited. The use of CRISPR-Cas9 has only been reported for genome editing in *Paenibacillus polymyxa* (17, 18), and only thymidylate synthase-based knockout systems have been reported for *P. elgii* B69 (19). Therefore, the development of novel gene editing tools for *Paenibacillus* is of great significance. Targetron, a gene targeting technology based on the group II intron system, comprises a group II intron RNA and a reverse transcriptase that recognizes target sites on double-stranded genomic DNA through base pair complementarity; this technology is inserted specifically into the target gene locus through a homing mechanism facilitated by reverse transcriptase, thereby inactivating the DNA (20, 21). The design and operation of Targetron technology for target sites are straightforward and efficient, and Targetron has been employed in the gene editing of various prokaryotes, including *Lactococcus lactis*, *Clostridium*, and *Escherichia coli* (*E. coli*) (22–24). However, no successful cases of use of Targetron technology in *Paenibacillus* species have been reported to date.

This study established an efficient electroporation protocol for *P. elgii* 219 involving the optimization of seven critical factors, namely, the growth medium, recovery medium, growth phase of targeted bacteria, electric field strength, electroporation buffer, cell wall-weakening agents, and plasmid DNA quality. This ultimately led to the development of a highly efficient electroporation method for *P. elgii* 219, achieving a transformation efficiency of up to 10^6^ transformants/µg DNA. This study also constructed a Targetron gene editing system for *P. elgii* 219 through leveraging the group II intron system. Employing the optimized transformation method, the plasmid was successfully transferred into *P. elgii* 219, and a putative priming glycosyltransferase was deleted, thereby eliminating the formation of viscous flocculent precipitates in the fermentation broth.

## MATERIALS AND METHODS

### Bacterial strains, plasmids, primers, and growth conditions

The bacterial strains and plasmids used in this study are listed in Table 1. *P. elgii* 219 was cultured in nutrient broth (NB; Sangon Biotech Co., Ltd., Shanghai, China) at 37°C. *E. coli* strains were grown in Luria–Bertani broth (LB; 10  g·L^−1^ sodium chloride, 10  g·L^−1^ tryptone, and 5  g·L^−1^ yeast extract). When necessary, 100 µg/mL of ampicillin (Sangon Biotech Co., Ltd., Shanghai, China) or 25 µg/mL of erythromycin (Sangon Biotech Co., Ltd., Shanghai, China) was added to the culture medium. In addition, the *E. coli* MC1061 strain was employed for plasmid construction and regular plasmid extraction. Plasmids from *E. coli* were purified using the SanPrep Column Plasmid Mini-Preps Kit (Sangon Biotech Co., Ltd., Shanghai, China) according to the manufacturer’s instructions.

**TABLE 1:** Strains and plasmids

Strains and plasmids	Relevant properties	Source
*E. coli* MC1061	Cloning strain	Our laboratory
*P. elgii* 219	Expression strain	Our laboratory
*P. elgii* 219 Δ4383	Expression strain	Our laboratory
pWAe	Expression plasmid, AmpR, and ErmR	This paper
pUC57	Cloning plasmid, group II intron	This paper
pDX4383	Gene modification, group II intron	This paper

### Initial transformation protocol

To obtain electrocompetent cells, a single clone of *P. elgii* 219 was cultured in 3 mL of NB media at 37°C and 220 rpm overnight (16 hours). Then, *P. elgii* 219 was transferred to a new NB medium (50 mL) and cultured under the same conditions for 24 hours. The saturated culture was transferred to a new NB medium (50 mL) with a 2% inoculum size and cultivated until the optical density (OD) at 600 nm reached 0.7 (OD_600_ = 0.7, detected using a spectrophotometer). The cells were cooled on ice for 15 minutes and collected via centrifugation at 4°C and 4000 × *g* for 15 minutes. All subsequent operations for preparing receptive cells were strictly conducted on ice. After being washed three times with ice-cold electroporation buffer (10% sucrose +1 mM MgCl_2_ (SM solution)) containing 100%, one-half, or one-fourth of the original medium volume, the electrocompetent cells were resuspended in 1/100 vol of the original culture (concentrated 100-fold). Aliquots of 100 µL of electrocompetent cells were mixed with 100 ng of the plasmid (100 ng/µL) and incubated on ice for 10 minutes. Then, the mixture was transferred to a 1 mm cuvette (Bio-Rad, Hercules, CA, USA), and an electric pulse was applied at 18 kV/cm^−1^. The cells were immediately diluted with 900 µL of the NB medium, shaken at 37°C and 220 rpm for 3 hours, and harvested and plated on NB agar plates supplemented with 25 µg/mL erythromycin. After 36 hours of incubation at 37°C, the transformants were counted, and the transformation efficiency (transformants/μg DNA) was calculated.

### Protocol optimization

#### Growth medium optimization

To identify a suitable medium for cell culture, *P. elgii* 219 was cultured in the following media: NB, LB, brain–heart infusion (BHI; Beijing Solarbio Science & Technology Co., Ltd., China), King’s broth (KB; 20 g/L tryptone, 1.15 g/L K_2_HPO_4_, 1.5 g/L MgSO_4_·7H_2_O, and 1.5% glycerol) (25), and Muller–Hinton broth (MH; Beijing Solarbio Science & Technology Co., Ltd., China). Subsequent steps were performed following the initial protocol described above.

#### Cell collection phase

To further investigate the effects of different collection stages on the transformation of electrocompetent cells, the growth curve of *P. elgii* 219 in the KB medium was measured. *P. elgii* 219 was cultured in KB media and collected when the culture reached OD_600_ values of 0.1, 0.3, 0.5, 0.7, 0.9, 1.5, 3.0, and 4.0, with OD_600_ values of 1.5, 3.0, and 4.0 corresponding to the early, middle, and late phases of the logarithmic period, respectively. Subsequently, electrocompetent cells were prepared as described above.

#### Electric field optimization

Different species of bacteria vary widely in terms of their requirements for electric transfer voltage. To explore the optimal electric pulse for electrocompetent cells, the electric field strength of the electroporation was measured at 9, 12, 15, 18, 21, and 24 kV cm^−1^.

#### Washing solution optimization

According to the results of the above experiments, the culture and collection procedures were optimized as follows: bacteria were cultured to a specific stage in KB, cells were collected in an ice bath for 15 minutes, electrocompetent cells were prepared using different electroporation buffers (Table 2), and cells were resuspended in the same buffer. The electroporation procedure was set to the optimal field strength determined using the test described above. Subsequent experiments were performed as described in “Initial transformation protocol,” above.

**TABLE 2:** Electroporation buffer composition

Solution	Composition
SM	10% sucrose +1 mM MgCl_2_
SMGG	SM +10% glycerol
SMH	SM +1 mM HEPES
SMP	SM +1 mM phosphate-buffered saline (PBS) (pH = 7.2–7.4)
SMAG	0.5 M sorbitol +0.5 M mannitol +10% glycerol
SMHP	0.25% M sucrose +1 mM MgCl_2_ +1 mM HEPES +1 mM PBS (pH = 7.2–7.4)
10%G	10% glycerol
S5M	5% sucrose +1 mM MgCl_2_
S15M	15% sucrose +1 mM MgCl_2_
SM0	10% sucrose
SM2	10% sucrose +2 mM MgCl_2_

#### Recovery medium optimization and recovery time

To determine the optimal recovery medium for *P. elgii* 219 after electroporation, NB, LB, KB, MH, BHI, tryptic soy broth (TSB), and super optimal broth with catabolic repressor (SOC) media were evaluated. Following the electroporation process, 900 µL of the recovery culture medium was added. To evaluate the effect of the resuscitation time on cell recovery and plasmid transformation, the recovery times were set to 1 hour, 3 hours, and 16 hours.

#### DNA optimization

The preparation of plasmid pWAe is described above. In the final determination of optimized DNA content, electroporation was performed with a 100 µL equal sample mixed with different amounts of DNA (25, 50, 100, 250, 500, 750, and 1,000 ng plasmid pWAe).

#### Cell wall weakening

Glycine, DL-threonine, isonicotinic acid hydrazide (INH), Tween 80, and ampicillin were selected as cell wall-weakening agents (all reagents were purchased from Sangon Biotech Co., Ltd., Shanghai, China). According to the above results, *P. elgii* 219 was cultured in KB at an OD_600_ of 0.5, and the cell wall-weakening agents such as glycine (2%, 4%, 6%, and 8%, wt/vol, final concentration), DL-threonine (2%, 4%, 6%, and 8%, wt/vol, final concentration), INH (0.2%, 0.4%, 0.6%, and 0.8%, wt/vol, final concentration), Tween 80 (0.2%, 0.4%, 0.6%, and 0.8%, wt/vol, final concentration), and ampicillin (2.5%, 5%, 7.5%, and 10%, wt/vol, final concentration) were added to the culture media and incubated for 1 hour. Following incubation, the cells were collected to prepare the electrocompetent cells.

### Plasmid construction and genome editing

To identify genes involved in EPS biosynthesis, the *P. elgii* 219 genome was uploaded to RAST for automated genome annotation. The nucleotide sequence and the corresponding deduced proteins were analyzed using a BlastP homology search, and *gene_4383* was mapped to the *P. elgii* 219 genome (PRJNA1193137), revealing that it was 99% similar to a priming glycosyltransferase (WP_054974698) from *Paenibacillus* sp. A3 (Table S2), which catalyzes the transfer of a sugar from a donor to a lipid carrier, such as undecaprenyl phosphate.

**Fig 1:**
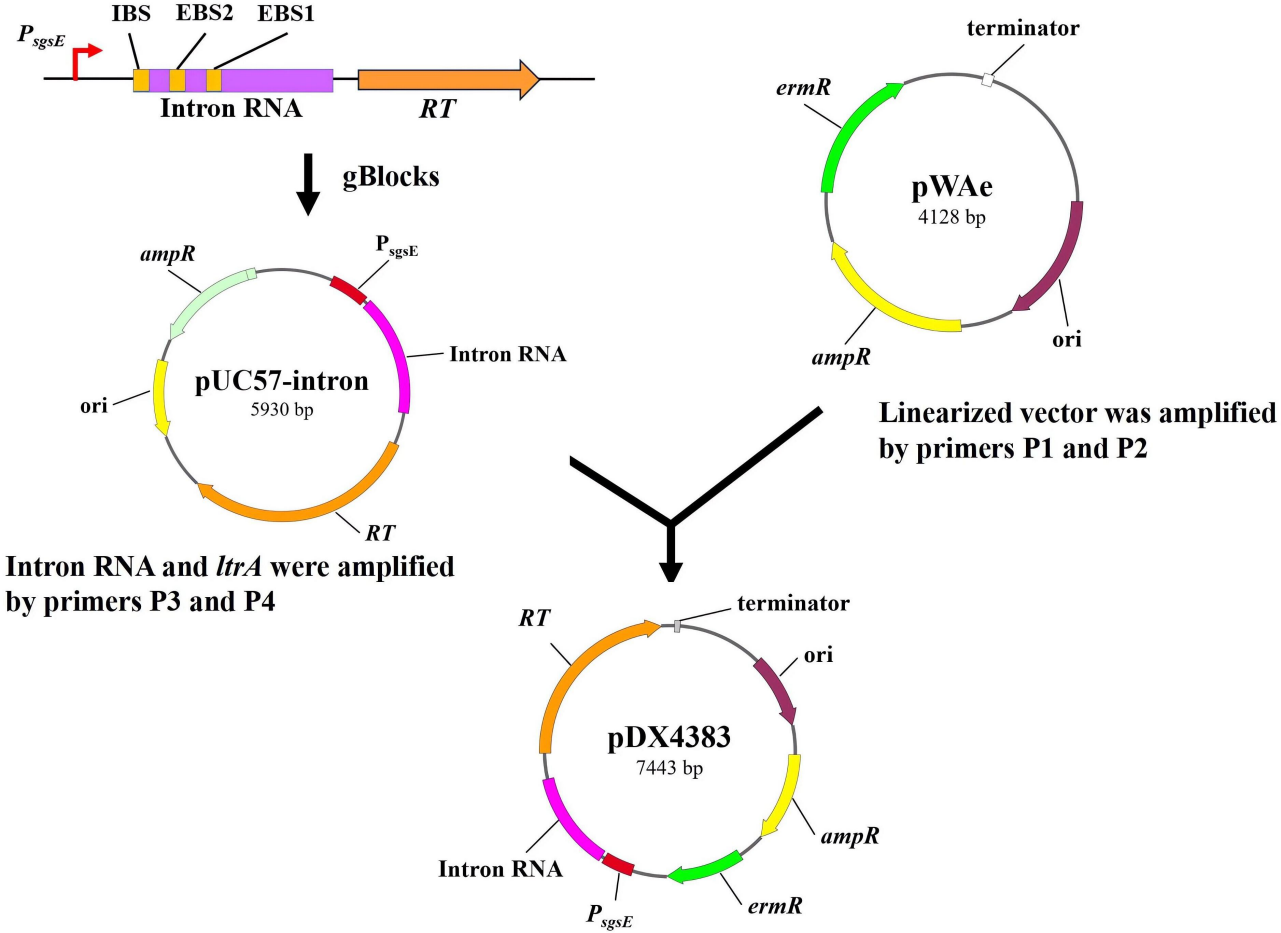
Basic plasmid pWAe and construction of the Targetron vector pDX4383 in *Paenibacillus elgii* 219. The target sites of *gene_4383* were designed using an online tool (www.clustron.com), including the intron-binding site (IBS), exon-justice chain binding site 2 (EBS2), and exon-antisense chain binding site 1 (EBS1). Abbreviations: ori, origin of replication; *ampR*, ampicillin resistance; *ermR*, erythromycin resistance; *P_sgsE_*, promoter *sgsE; RT*, reverse transcriptase.

To construct the pDX4383 plasmid (Fig. 1), the linearized vector pWAe was amplified using the primers P1 and P2. The sgsE promoter (26) and intron II sequence were obtained as artificial gBlocks (Sangon Biotech Co., Ltd., Shanghai, China), and the targeted region was designed using an online retargeting algorithm (www.clostron.com), including the intron-binding site (IBS), exon-justice chain binding site 2 (EBS2), and exon-antisense chain binding site 1 (EBS1). The fragment included the promoter *sgsE*, and the group II intron box was amplified with primers P3 and P4. All primers and DNA information used are listed in Table S1. Two DNA fragments were assembled via Gibson assembly and verified based on sequencing (Sangon Biotech Co., Ltd., Shanghai, China).

### Statistical analysis

Two-tailed Student’s *t-*tests were employed for single comparisons, while analysis of variance (ANOVA) with Tukey’s tests were utilized for multiple comparisons. *P* < 0.05 was considered statistically significant. Statistical analysis and graphing were performed using GraphPad Prism 9 software. Data are presented as the mean ± standard error of the mean (SEM), unless otherwise stated.

## RESULTS

### Culture medium optimization

**Fig 2:**
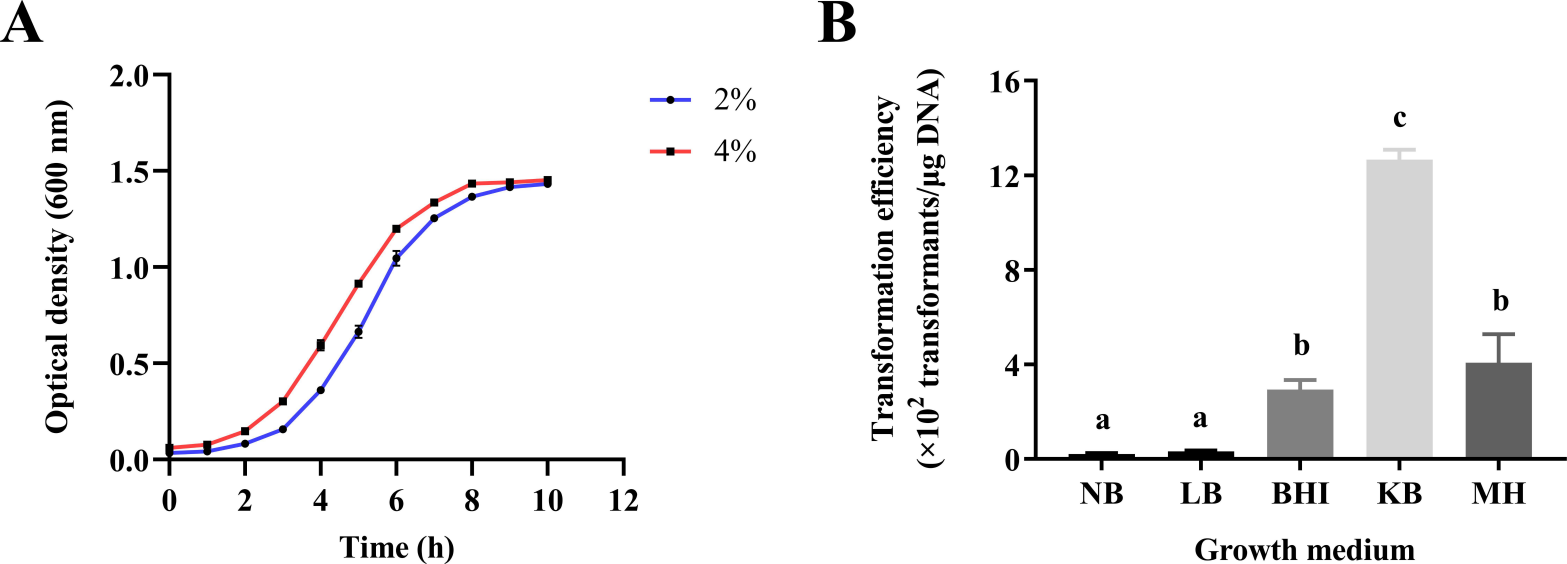
(**A**) Growth curve and transformation efficiencies of *Paenibacillus elgii* 219 cultured in the NB medium at 37°C and 220 rpm. The OD_600_ value of fermentation solution per hour was determined. Blue and red lines represent the growth curves of *P. elgii* 219 at inoculation amounts (vol/vol) of 2% and 4%, respectively. Each point on the curve represents the average of three replicates. (**B**) Transformation efficiencies obtained when preparing electrocompetent cells from *P. elgii* 219 using different culture media. *P. elgii* 219 cells were collected at an OD_600_ = 0.7, washed with 10% sucrose +1 mM MgCl_2_ (SM) solution, concentrated to 1/100 of the initial volume, and mixed with 100 ng of pWAe before applying an electrical pulse of 18 kV cm^−1^. All cells were resuscitated in the NB medium at 37°C. All experiments were independently repeated three times. NB: nutrient broth medium; LB: Luria–Bertani medium; BHI: brain–heart infusion; KB: King’s broth medium; MH: Müller–Hinton medium. Different lowercase letters indicate significant differences between treatments (*P* < 0.05).

Selecting the appropriate growth medium is an important factor affecting the bacterial transformation efficiency. To screen for an appropriate growth medium, *P. elgii* 219 was cultured in five media (NB, LB, BHI, KB, and MH) for the preparation of electrocompetent cells. As shown in Fig. 2B, the NB medium as a standard medium resulted in a low transformation efficiency (0.22 × 10^2^ transformants/μg DNA), while bacteria cultured in BHI and MH media achieved a high transformation efficiency (2.94 × 10^2^ transformants/μg DNA and 4.08 × 10^2^ transformants/μg DNA, respectively). Surprisingly, the transformation efficiency was greatly improved when using KB media, exceeding 1.27 × 10^3^ transformants/μg DNA. This study further examined the growth performance of *P. elgii* 219 across various media. As depicted in Fig. S1, *P*. *elgii* 219 achieved a higher OD value when cultured in the KB medium, followed by BHI and MH media. In contrast, *P. elgii* 219 attained lower OD values in NB and LB media.

Thus, KB medium proved to be a suitable medium for *P. elgii* 219 and was therefore employed to prepare electrocompetent cells in subsequent tests.

### Impact of the cell collection stage on the transformation efficiency

**Fig 3:**
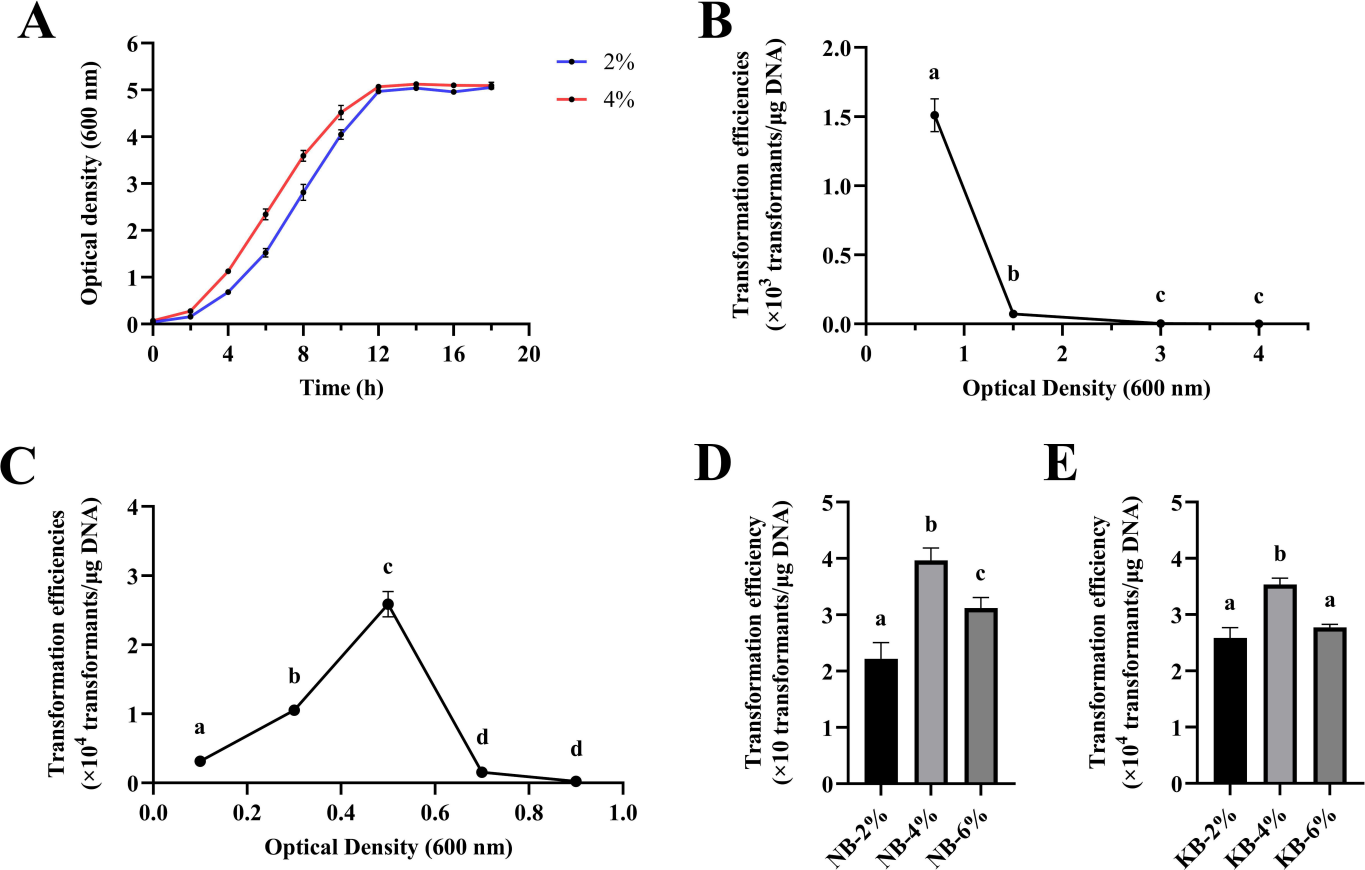
Effects of collection stages and inoculation amount on the transformation efficiency of *Paenibacillus elgii* 219. (**A**) Growth curve of *P. elgii* 219 in King’s broth (KB) medium. Blue and red lines represent the growth curve of *P. elgii* 219 at inoculation amounts (vol/vol) of 2% and 4%, respectively. Each point on the curve represents the average of three replicates. (**B, C**) Electroporation efficiencies of competent cells from different stages. (**D, E**) Different inoculations affected the transformation efficiency of cells harvested with nutrient broth (NB) and KB medium. Electrocompetent cells from *P. elgii* 219 were harvested at different growth stages. The cells were washed with 10% sucrose +1 mM MgCl_2_ (SM) solution, concentrated to 1/100 of the initial volume, and mixed with 100 ng of pWAe before applying an electric pulse of 18 kV cm^−1^. All cells were resuscitated in the NB medium at 37°C. All experiments were independently repeated three times. Different lowercase letters indicate significant differences between treatments (*P* < 0.05).

To investigate the effects of the stage at which the cells were harvested on the transformation efficiency, the growth curve of *P. elgii* 219 in KB medium was examined (Fig. 3A). Electrocompetent cells prepared from *P. elgii* 219 in the early, middle, and late phases of logarithmic growth (OD_600_ = 1.5, 3, and 4, respectively) produced only a limited number of transformants (Fig. 3B). Consequently, the cells were harvested at OD_600_ = 0.1, 0.3, 0.5, 0.7, and 0.9 to prepare the electrocompetent cells. As a result (Fig. 3C), a significant improvement in the protocol’s transformation efficiency was achieved when *P. elgii* 219 grew to an OD_600_ of 0.5 (increased to 2.59 × 10^4^ transformants/μg DNA), which corresponded to the early-log growth phase. Other growth phases yielded poor efficiencies.

In addition, different inoculations had significant effects on the growth rate of *P. elgii* 219 (Fig. 2A and 3A). Therefore, this study further examined the effects of *P. elgii* 219 under different inoculations for the preparation of receptive cells. Cells harvested from 4% (vol/vol) inoculation volume exhibited a higher transformation efficiency in both NB (Fig. 3D) and KB (Fig. 3E) media.

### Electroporation pulse electric field intensity

**Fig 4:**
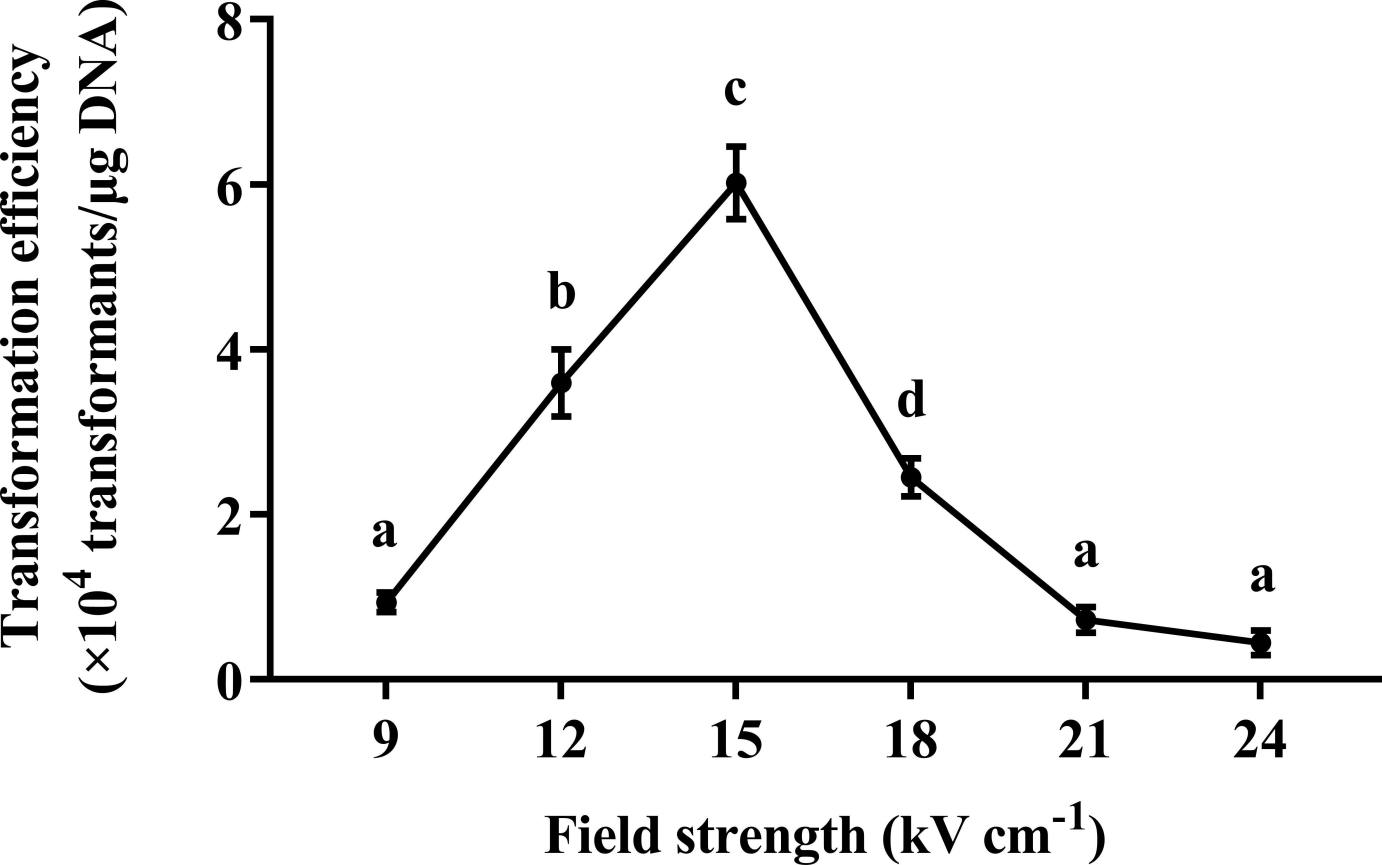
Effect of field strength on the transformation efficiency of *Paenibacillus elgii* 219. *P. elgii* 219 was cultured in King’s broth (KB) medium with 4% (vol/vol) inoculation volume and harvested at OD_600_ = 0.5. The cells were washed with 10% sucrose +1 mM MgCl_2_ (SM) solution, concentrated to 1/100 of the initial volume, and mixed with 100 ng of pWAe before applying an electrical pulse of varying field strength. All cells were resuscitated in the nutrient broth (NB) medium at 37°C. All experiments were independently repeated three times. Different lowercase letters indicate significant differences between treatments (*P* < 0.05).

Due to differences in size, morphology, and characteristics, bacteria have different requirements for electric field strength. Therefore, to optimize the electric field strength of *P. elgii* 219, a field intensity gradient (9–24 kV cm^−1^) was employed to prepare the electrocompetent cells. As shown in Fig. 4, in this protocol, the optimal electric field intensity was 15 kV/cm^−1^, which allowed the transformation efficiency to reach 6.02 × 10^4^ transformants/μg DNA. Therefore, this value was used in subsequent experiments. In addition, the electroporation transformation efficiency significantly declined after either an increase or a decrease in the voltage.

### Electroporation buffer

**Fig 5:**
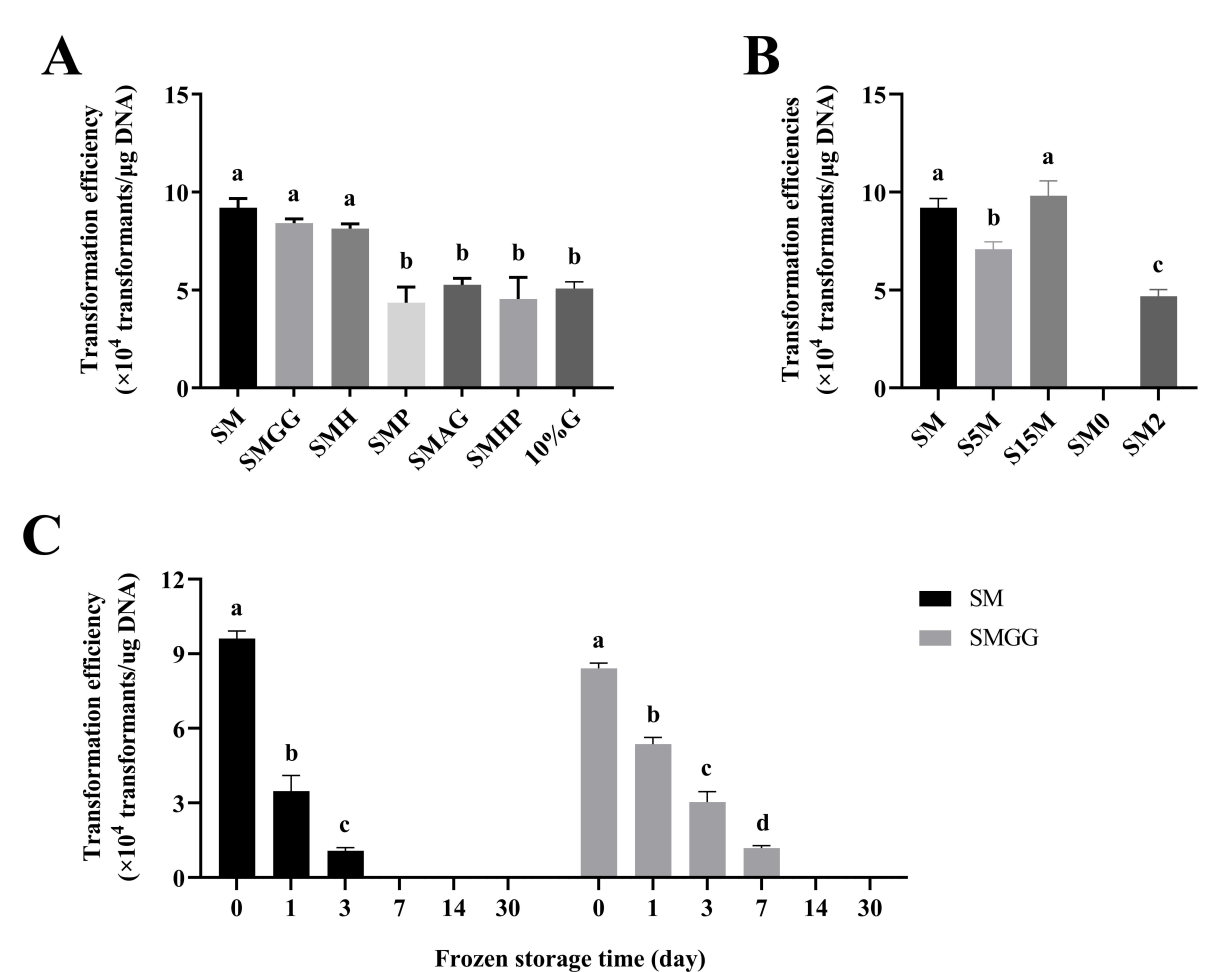
(A and B) Effects of the electroporation buffer and (C) cryopreservation time on the transformation efficiency of *Paenibacillus elgii* 219. *P. elgii* 219 was cultured in King’s broth (KB) medium with 4% (vol/vol) inoculation volume and harvested at OD_600_ = 0.5. The cells were washed with different solutions, concentrated to 1/100 of the initial volume, and mixed with 100 ng of pWAe before applying an electric pulse of 15 kV/cm^−1^. All cells were resuscitated in the nutrient broth (NB) medium at 37°C. All experiments were independently repeated three times. Different lowercase letters indicate significant differences between means (*P* < 0.05). Abbreviations: SM, 10% sucrose +1 mM MgCl_2_; SMGG, SM + 10% glycerol; SMH, SM + 1 mM 4-(2-hydroxyethyl)−1-piperazineëthanesulfonic acid (HEPES); SMP, SM + 1 mM phosphate-buffered saline (PBS) (pH = 7.2–7.4); SMAG, 0.5 M sorbitol +0.5 M mannitol +10% glycerol; SMHP, 0.25% M sucrose +1 mM MgCl_2_ +1 mM HEPES +10% glycerol; 10%G, 10% glycerol; S5M, 5% sucrose +1 mM MgCl_2_; S15M, 15% sucrose +1 mM MgCl_2_; SM0, 10% sucrose; SM2, 10% sucrose +2 mM MgCl_2_.

A total of seven electroporation buffers [SM; SM +10% glycerol (SMGG); SM +4-(2-hydroxyethyl)−1-piperazineethanesulfonic acid (HEPES) (SMH); SM +phosphate-buffered saline (PBS) (SMP); 0.5 M sorbitol +0.5 M mannitol +10% glycerol (SMAG); 0.25% M sucrose +1 mM MgCl_2_ +1 mM HEPES +1 mM PBS at pH = 7.2–7.4 (SMHP); and 10% glycerol (10% G)] (Table 2) were evaluated. The results (Fig. 5A) showed that the best transformation efficiency was obtained using the SM solution (containing 10% sucrose and 1 mM MgCl_2_). Previous research demonstrated that adding HEPES or PBS to electroporation solutions increased the transformation efficiency (9). However, the addition of HEPES (SMH solution) or PBS (SMP solution) did not have a positive effect in the present study. Similarly, the transformation efficiency was not improved when using 10% G or SMAG. This study also evaluated the effects of sucrose and magnesium chloride concentrations on the transformation efficiency. When the sucrose concentration was reduced from 10% to 5% (SM vs S5M), a significant decrease in the transformation efficiency was observed, while an increase in sucrose to 15% (S15M) seemed to have no effect (Fig. 5B). Additionally, sucrose solutions lacking magnesium chloride (SM0) were unable to generate transformants, and an increase in magnesium chloride to 2 mM MgCl_2_ (SM2) did not appear to be conducive to electroporation (Fig. 5B).

Based on the effect of glycerol on cell cryopreservation, electrocompetent cells were prepared using SM and glycerin-supplemented buffer (SMGG) and frozen at −80°C. The results (Fig. 5C) demonstrated that the addition of glycerol could prolong the transformation ability of the electrocompetent cells, but the transformation ability decreased markedly as the freezing time was extended. No transformants were obtained from competent cells stored at −80°C for 1 month.

### Optimizing the recovery medium and duration

**Fig 6:**
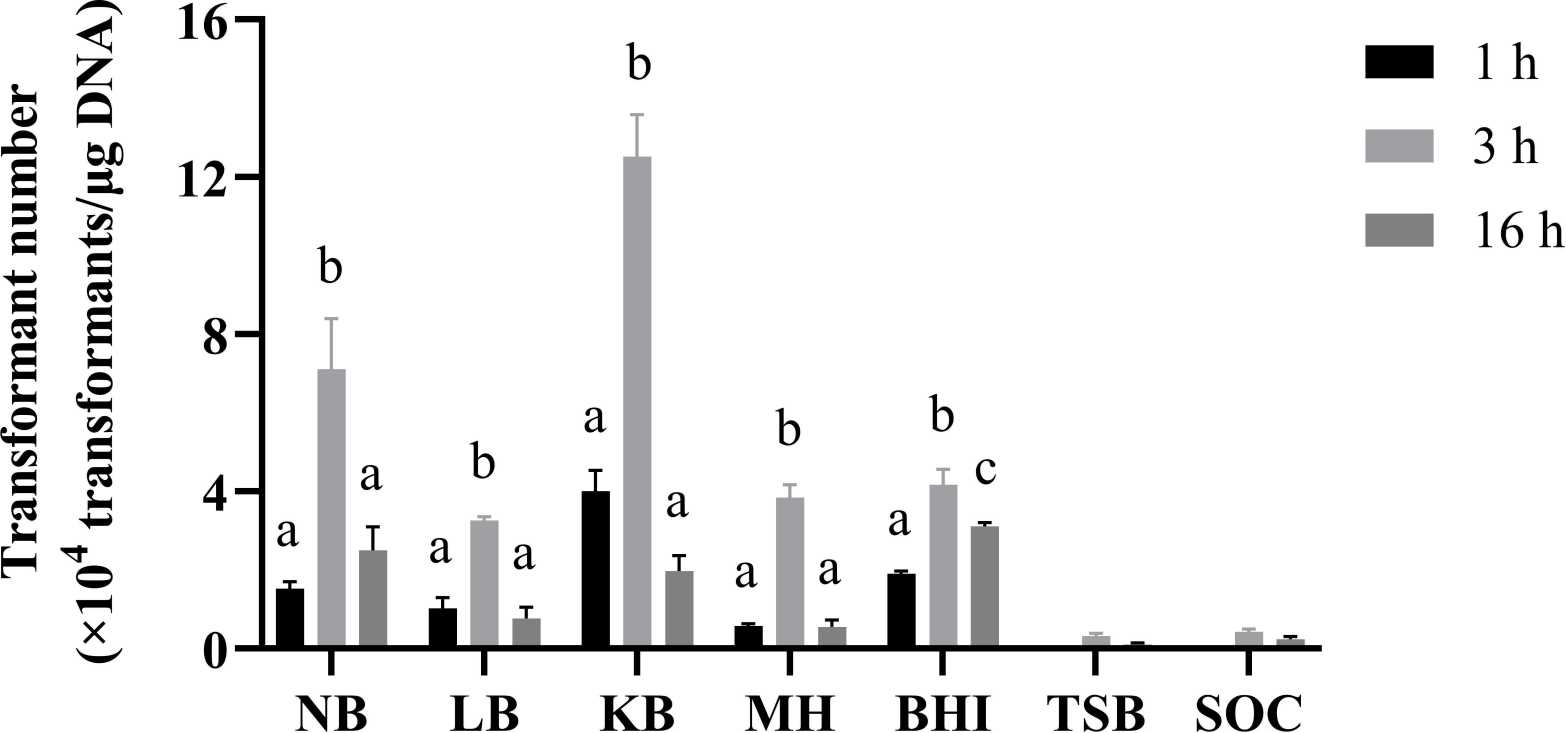
Effect of recovery medium and time on the transformation efficiency of *Paenibacillus elgii* 219. *P. elgii* 219 was cultured in King’s broth (KB) medium with 4% (vol/vol) inoculation volume and harvested at OD_600_ = 0.5. The cells were washed with 15% sucrose +1 mM MgCl_2_ (S15M) solution, concentrated to 1/100 of the initial volume, and mixed with 100 ng of pWAe before applying an electric pulse of 15 kV cm^−1^. All cells were resuscitated in different media at 37°C. All experiments were independently repeated three times. NB: nutrient broth medium; LB: Luria–Bertani medium; BHI: brain–heart infusion; MH: Müller–Hinton medium; TSB, tryptic soy broth; SOC, super optimal broth with catabolic repressor. Different letters indicate significant differences between means (*P* < 0.05).

After electroporation, the cells were subjected to membrane repair and plasmid expression. Seven growth media were tested as recovery media (NB, LB, KB, MH, BHI, SOC, and TSB). Interestingly, KB was the best medium for the preparation of *P. elgii* 219 electrocompetent cells (Fig. 6), and the transformation efficiency reached 1.25 × 10^5^ transformants/μg DNA.

The recovery time has an important effect on cell repair and plasmid expression. Therefore, three recovery times of 1 hour, 3 hours, and 16 hours were employed for comparison. The results showed that more transformants were obtained at 3 hours of resuscitation, while limited transformants were produced at 1 hour and 16 hours of resuscitation (Fig. 6).

### Optimizing DNA quantity and quality

**Fig 7:**
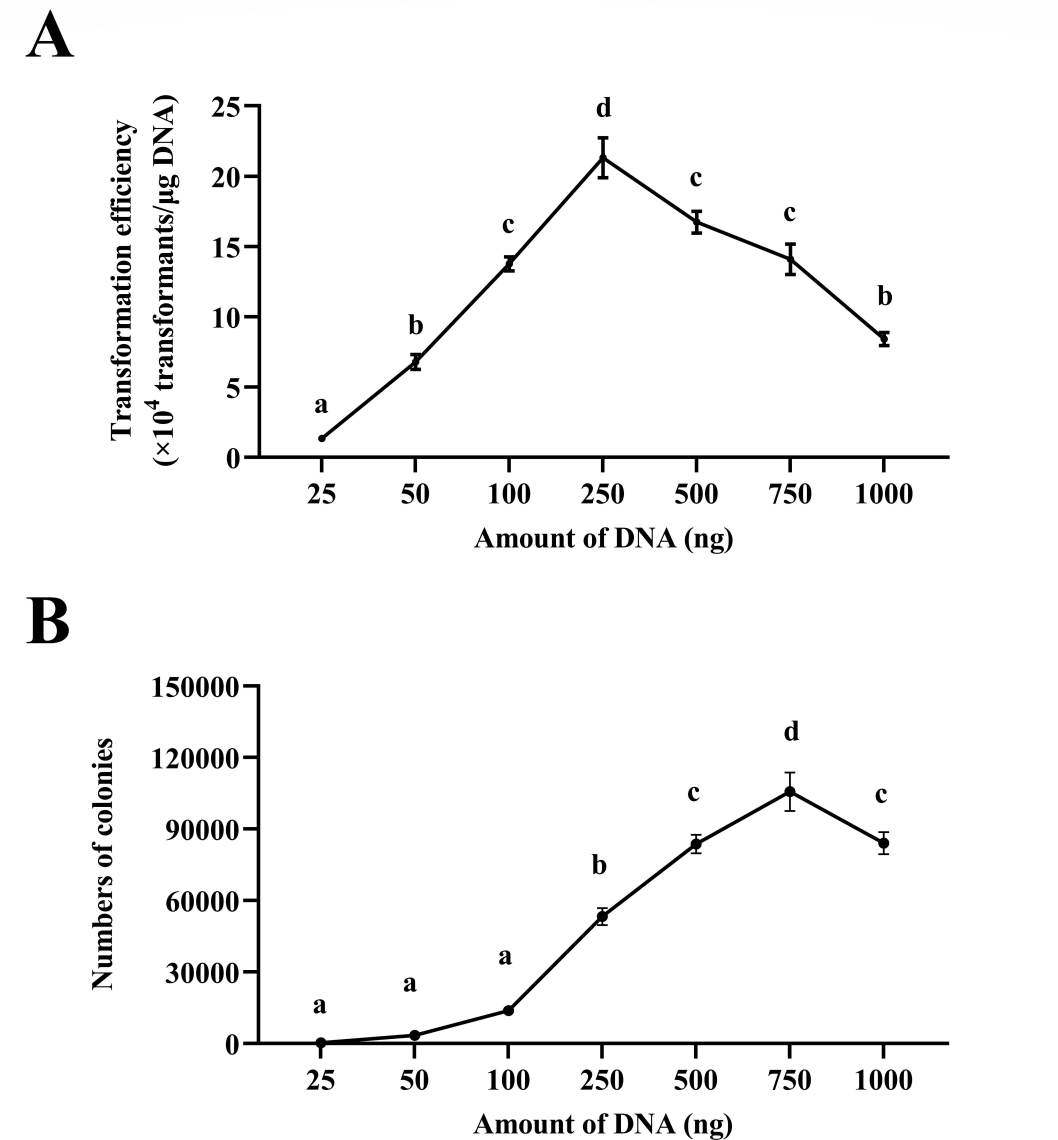
Effect of DNA quantity on the transformation efficiency. *Paenibacillus elgii* 219 was cultured in King’s broth (KB) medium with 4% (vol/vol) inoculation volume and harvested at OD_600_ = 0.5. The cells were washed with 15% sucrose +1 mM MgCl_2_ (S15M) solution and concentrated to 1/100 of the initial volume and mixed with pWAe (25–1000 ng) before applying an electric pulse of 15 kV cm^−1^. All cells were resuscitated in the KB medium at 37°C. All experiments were independently repeated three times. Different lowercase letters indicate significant differences between means (*P* < 0.05).

The effect of the amount of the plasmid on the electroporation transformation efficiency was further evaluated. As shown in Fig. 7A, compared to the initial protocol, the transformation efficiency increased to 2.13 × 10^5^ transformants/μg DNA when 250 ng of plasmid was added. Furthermore, despite a decrease in the transformation efficiency, adding more plasmids resulted in more colonies (Fig. 7B). Notably, the peak number of transformants (10.57 × 10^4^) was achieved with the addition of 750 ng of plasmid (Fig. 7B).

### Cell wall weakening

**Fig 8:**
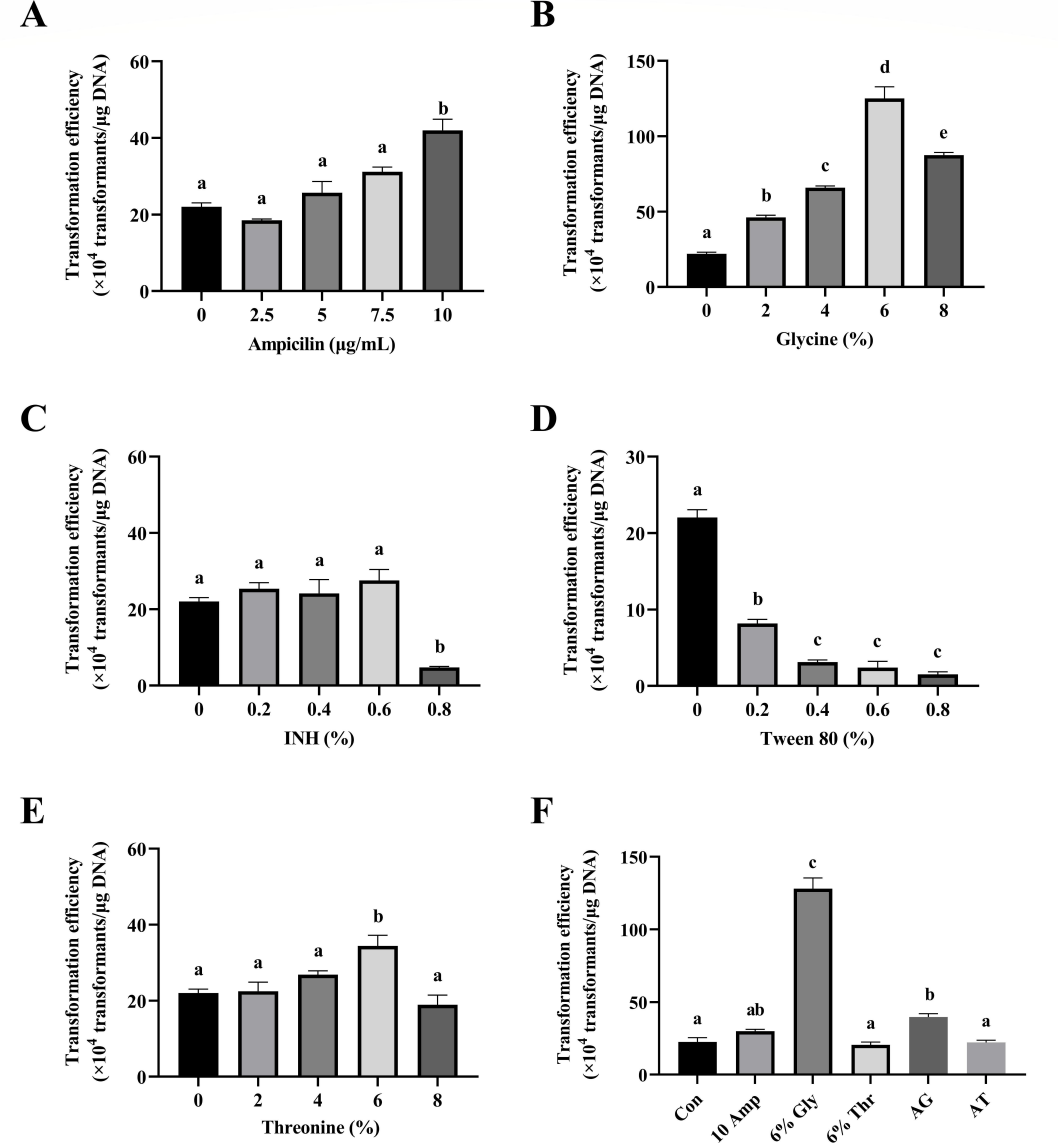
Effects of cell wall-weakening agents on the transformation efficiency of *Paenibacillus elgii* 219. *P. elgii* 219 was cultured in King’s broth (KB) medium with 4% (vol/vol) inoculation volume until OD_600_ = 0.5, and then cell wall-weakening agents were added and cultured for another 1 hour. The cells were harvested and washed with 15% sucrose +1 mM MgCl_2_ (S15M) solution, concentrated to 1/100 of the initial volume, and mixed with 250 ng of pWAe before applying an electric pulse of 15 kV/cm^−1^. All cells were resuscitated in the KB medium at 37°C. All experiments were independently repeated three times. Con, no cell wall-weakening agent was added; 10 Amp, 10 µg/mL ampicillin was added; 6% Gly, 6% glycine was added; 6% Thr, 6% threonine was added; AG, 10 µg/mL ampicillin and 6% glycine were added; AT, 10 µg/mL ampicillin and 6% threonine were added. Different lowercase letters indicate significant differences between means (*P* < 0.05).

In gram-positive bacteria, the cell wall hinders the entry of foreign DNA and results in a lower electroporation transformation efficiency compared to gram-negative bacteria (27). The use of cell wall-weakening agents is an effective means of improving the efficiency of gram-positive bacteria point transformation. To investigate the effect of cell wall weakening on *P. elgii* 219, four cell wall-weakening agents were employed to prepare competent cells. As depicted in Fig. 8, the transformation efficiencies of ampicillin, glycine, threonine, and INH were enhanced. The use of 10 µg/mL ampicillin, 6% threonine, and 0.6% INH yielded efficiencies of 4.20 × 10^5^ transformants/μg DNA, 3.44 × 10^5^ transformants/μg DNA, and 2.76 × 10^5^ transformants/μg DNA, respectively. Interestingly, the best transformation efficiency was obtained using 6% glycine, with the transformation efficiency reaching 1.25 × 10^6^ transformants/μg DNA. However, the addition of Tween 80 resulted in a significant reduction in the transformation efficiency. Furthermore, the use of cell wall-weakening agents inhibited the growth of *P. elgii* 219 (Fig. S3).

Based on the mechanisms of cell wall-weakening agents, ampicillin and INH, glycine, and threonine were employed in combination to prepare electrocompetent cells. As shown in Fig. 8F, combinations of 10 µg/mL ampicillin with 6% glycine (AG) and 10 µg/mL ampicillin with 6% threonine (AT) did not further enhance the transformation efficiency.

### Protocol application: glycosyltransferase inactivation

The optimized protocol was as follows: *P. elgii* 219 was cultured in the KB medium to OD_600_ = 0.5, and then 6% glycine was added and cultured for a further 1 hour. The cells were harvested and washed with S15M solution, concentrated to 1/100 of the initial volume, and mixed with 250 ng of pWAe before applying an electric pulse of 15 kV/cm^−1^. Cells were resuscitated in the KB medium at 37°C and 220 rpm for 3 hours and cultured on NB agar plates supplemented with 25 µg/mL erythromycin.

Using the optimized conditions (Table 3), the transformation efficiency was improved to 1.25 × 10^6^ transformants/μg DNA. A case study was employed to evaluate the transformation efficiency. To verify the efficiency of the electroporation protocol, the Targetron knockout plasmid pDX4383 was constructed, and 2.36 × 10^2^ transformants were obtained.

**TABLE 3:** Electroporation optimized protocol and transformation efficiency

Optimized conditions	Results	Transformation efficiency (transformants/μg DNA)
Initial protocol		0.22 × 10^2^
Growth medium	KB	1.27 × 10^3^
Cell collection phase	OD_600_ = 0.5	2.59 × 10^4^
Inoculum volume	4%	3.54 × 10^4^
Field strength	15 kV cm^−1^	6.02 × 10^4^
Electroporation buffer	15% sucrose, 1 mM MgCl_2_	9.81 × 10^4^
Recovery medium and time	KB, 3 h	1.25 × 10^5^
DNA quantities	250 ng	2.13 × 10^5^
Cell wall-weakening agent	6% glycine	1.25 × 10^6^

**Fig 9:**
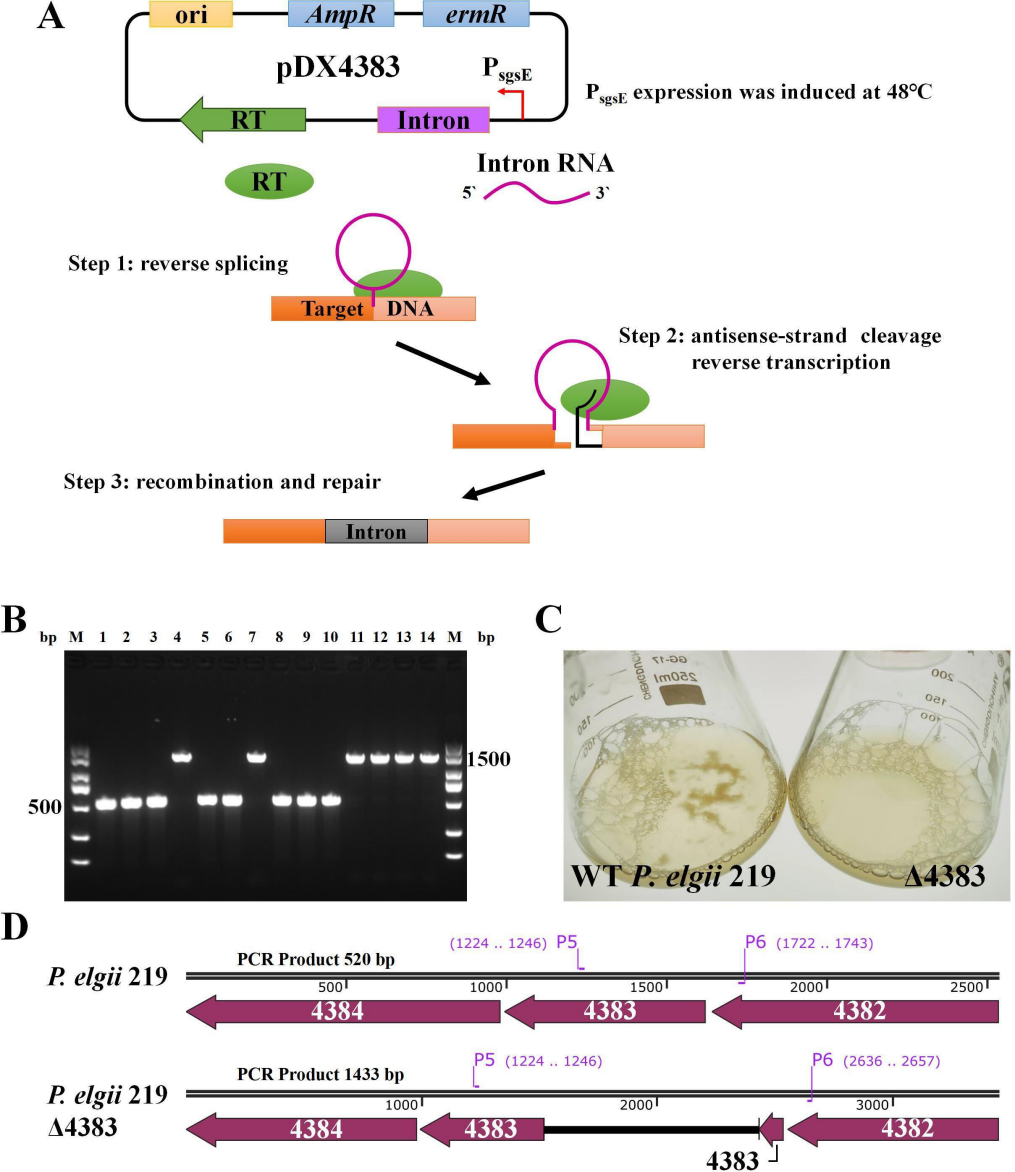
Inactivation of the putative guide glycosyltransferase using Targetron. (**A**) Schematic map of the knockout strategy. The intron RNA recognizes and cleaves the DNA target site through base-pairing complementarity, while reverse transcriptase (RT) assists in the splicing and integration of the intron RNA through stabilizing the active RNA structure. Furthermore, the intron-encoded protein possesses reverse transcriptase activity, enabling it to use the inserted intron RNA as a template to synthesize complementary DNA via reverse transcription, thereby integrating the intron RNA into the DNA target site. (**B**) Inactivation of priming glycosyltransferase and (**C**) fermentation status of the mutant strain for 24 hours. (**D**) Chromosome region and primer annealing sites. A Targetron plasmid pDX4383 was transformed using an optimized electroporation protocol. Bands 1–14 indicate the polymerase chain reaction (PCR) results of amplifying the target *gene_4383* of *P. elgii* 219 and *P. elgii* 219 Δ4383 using primers P5 and P6. M, marker; Intron, group II intron insert region.

To inactivate the putative priming glycosyltransferase (*gene_4383*), a transformant was selected and cultured in the NB medium to the mid-logarithmic growth phase, incubated at 48°C for an additional 1 hour, diluted, and then spread onto plates supplemented with erythromycin (25 µg/mL). The Targetron working mechanism and insertion site are depicted in Fig. 9A and D. Fourteen transformants were randomly selected and verified using PCR. As shown in Fig. 9B, the mutants had 1,433 bp fragments (bands 4, 7, 11, 12, 13, and 14), as verified by PCR with the specific primers P5 and P6, which indicated that the intron sequence had been inserted into the targeted site of *gene_4383* (520 bp vs 1,433 bp). Compared to the wild-type *P. elgii* 219, the Δ4383 mutant strain exhibited no adhesion when subjected to shake flask fermentation in NB media for 24 hours (Fig. 9C).

### EPS elimination improves the efficiency of electroporation

**Fig 10:**
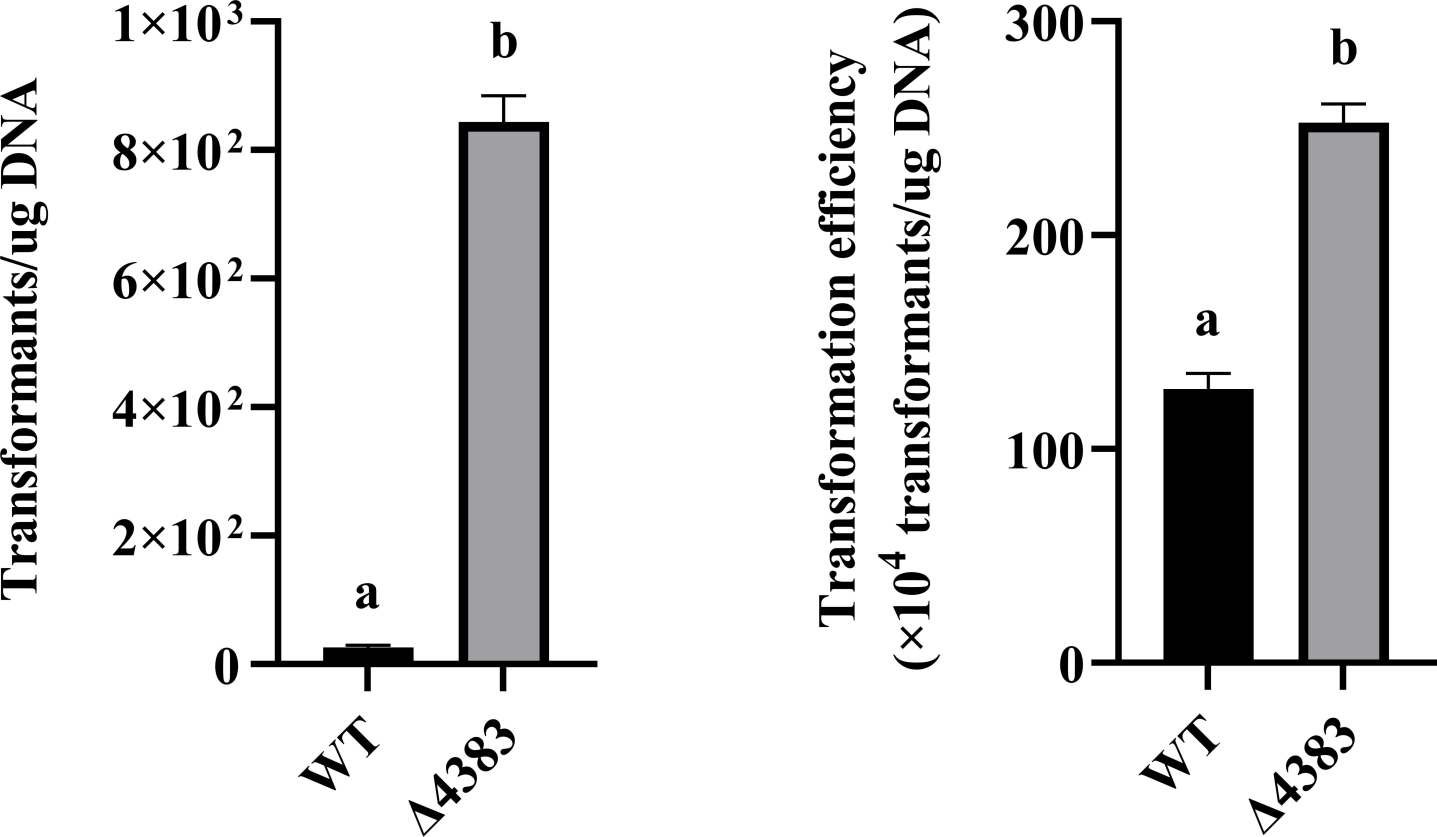
Electroporation transformation efficiency of *Paenibacillus elgii* 219 under the (**A**) initial and (**B**) optimized protocols after the deletion of exopolysaccharides (EPS). All experiments were independently repeated three times. WT, wild-type *P. elgii* 219; Δ4383, *P. elgii* 219 Δ4383.

Based on previous reports that the deletion of EPS can improve the transformation efficiency, this study compared the differences in electroporation transformation efficiency between wild-type *P. elgii* 219 and *P. elgii* 219 Δ4383. As shown in Fig. 10, under both the initial and optimized protocols, the mutant strain with the deletion of EPS achieved a higher transformation efficiency compared to that of the wild-type strain. After the removal of EPS, *P. elgii 219* Δ4383 exhibited an increased growth rate in NB and KB media and attained a higher OD_600_ value (Fig. S2).

## DISCUSSION

This study improved the ability of *P. elgii* 219 electrocompetent cells to transform exogenous plasmid DNA. The effects of the culture medium composition, growth stage, voltage at which electroporation was performed, cell-wall-weakening agents, electroporation buffer composition, and DNA quality on the transformation efficiency of *P. elgii* 219 electroporation were systematically evaluated. Using this method, the plasmid transformation efficiency of *P. elgii* 219 increased to 10^6^ transformants/μg DNA. This is the highest level of transformation efficiency reported for *Paenibacillus* to date. In addition, this study established a group II intron gene targeting system for *P. elgii* 219, which successfully eliminated the production of EPS.

The electroporation technique transiently increases the permeability of cell membranes through applying a high-intensity electric field, enabling cells to absorb exogenous DNA molecules from their surrounding environment. This method is typically approximately 10 times more efficient than chemical transformation (28). The capacity of competent cells to transform DNA varies with changes in media components, growth conditions, and growth stages, primarily due to the influence of these factors on cell wall and membrane structures (15).

Reports on the optimization of transformation media for *Bacillus* and *Paenibacillus* species have revealed that the nutrient levels in the medium exert varying effects on the cellular transformation capacity. For example, *Paenibacillus riograndensis* SBR5 is transformable in BHI media that enables sufficient growth (9), whereas *Bacillus amyloliquefaciens* TA208 yields more transformants in the nutrient-deficient NCM medium (17, 28, 29). In this study, *P. elgii* 219 was required to be grown in a nutrient-rich medium to produce more transformants. Interestingly, the KB medium was the best medium for the preparation of *P. elgii* 219 electrocompetent cells, potentially due to the sufficient growth of *P. elgii* 219 in the KB medium, as evidenced by its attainment of high OD_600_ values.

In general, growth-stable cells typically have a higher resistance to electrical pulse-induced perforation and are more difficult to transform than cells of logarithmic growth (30). Current reports vary in terms of the optimal growth stage for achieving the highest transformation efficiency. For example, *Bacillus thuringiensis*, *Bacillus cereus*, and *P. riograndensis* SBR5 achieve the best results during the early exponential growth phase (9, 31, 32), while *Bacillus subtilis* and *B. amyloliquefaciens* exhibit the optimal transformation efficiency during the late exponential growth phase (33, 34). In the present study, *P. elgii* 219 cells collected at the early exponential growth phase achieved the greatest transformation efficiency.

Surprisingly, the volume of the inoculum exerted a notable influence on the transformation efficiency of *P. elgii* 219. Specifically, when *P. elgii* 219 was cultivated with a 2% inoculum volume and reached an OD_600_ of 0.5, few flocculent precipitates emerged in the fermentation broth, which reflected the adhesion between the bacteria and the EPS. EPS production can hinder the entry of exogenous DNA into cells (34). When the inoculation rate was increased from 2 to 4%, the transformation efficiency was significantly improved (Fig. 3D), and limited or no flocculation precipitation was observed in the fermentation solution. The elimination of viscous EPS (Fig. 10) also supported this result, which is consistent with the previous research (35).

Gram-positive bacteria possess a cell wall composed of a large amount of peptidoglycans, which serves as a physical barrier to hinder the entry of exogenous DNA (36). Altering the composition and structure of the cell wall or membrane through chemical treatments is an effective method for improving the transformation efficiency (37, 38). In the present study, the use of these cell wall-/membrane-interfering agents resulted in higher transformation efficiency for *P. elgii* 219, especially with the use of glycine. However, it should be noted that these reagents can inhibit the growth of *P. elgii* 219 (Fig. S3). Furthermore, based on the different mechanisms through which various agents act on bacterial cell walls, the present study examined the effect of combining glycine and threonine with ampicillin for treatment. However, these combinations did not appear to improve the transformation rate, possibly due to the cytotoxic effects of the combination of the optimal concentrations of each chemical reagent on *P. elgii* 219.

Electroporation effectively increases the permeability of the cell membrane. Under the influence of an instantaneous strong electric field, the cell membrane and cell wall of the cells in the solution are punctured and become permeable. However, different types of bacteria exhibit different levels of tolerance to voltage, making it necessary to explore optimal conversion voltage settings. Generally, the probability of plasmids entering cells increases as the voltage rises (15), explaining the enhanced transformation efficiency observed when the voltage is increased from 9 to 15 kV cm^−1^. However, when the voltage exceeds a threshold value, it can cause cell lysis and death (39), which may account for the sudden decrease in the transformation efficiency of *P. elgii* 219 when the voltage surpasses 18 kV cm^−1^. While transient electric fields facilitate the entry of foreign DNA into the cell interior, they also cause the outflow of genetic material, leading to cell death (40, 41). The use of an electroporation buffer can effectively reduce electrical conductivity and arc generation during electroporation, while providing good osmotic pressure protection for cells after strong electric field treatment (30). In this study, the use of S15M resulted in the highest transformation efficiency for *P. elgii* 219, while SM2 increased the conductivity of the solution.

Cells subjected to electroporation require the recovery of their cell membrane and cell wall in a nutrient-rich medium. The present study demonstrated that KB medium was an excellent formulation for the immediate recovery of *P. elgii* 219 cells post-electroporation. Studies have reported that substances such as sucrose, sorbitol, and glycerol provide good osmotic pressure protection for cells following strong electric field treatment (31). Interestingly, the KB medium contains glycerol, which was the reason exposure to the KB medium prevented the loss of cytoplasm and the death of *P. elgii* 219 cells compared to other recovery media. Additionally, the duration of recovery time for cells after electroporation is also a factor that should be considered. Like most *Bacillus* and *Paenibacillus* species, electrocompetent cells of *P. elgii* 219 require 3 hours to recover and grow after electroporation before being transferred to selection plates (9, 34). As expected, the shorter recovery period (1 hour) yielded only a limited number of inverters, and the longer recovery period (3 hours) would allow the cells to recover and divide, potentially leading to the acquisition of more colonies. However, in comparison to the 1 hour period, we lack evidence indicating that the extra transformants obtained at 3 hours are independent. They could potentially be the result of the division of the independent transformants from the 1 hour period. Therefore, although the recovery time of 3 hours produced more transformants, the transformation efficiency possibly remains unchanged. Additionally, as the recovery time is extended, cells cultured in a nonselective (antibiotic-free) environment may gradually lose their plasmids, which could explain the limited number of transformants obtained at 16 hours.

In addition, it should be noted that the quantity of plasmid DNA was also a factor that affects the transformation efficiency. The results are consistent with the fact that the transformation efficiency usually increases with the amount of plasmid DNA added (9, 34, 36). However, it is important to note that although increasing the amount of plasmids added will decrease the transformation efficiency, more transformants will be produced (Fig. 7B), potentially maximizing the number of independent transformants.

### Conclusion

The present study described a systematic evaluation method for the electroporation optimization of the *Paenibacillus* species, focusing on optimizing the electroporation transformation efficiency of wild-type *P. elgii* 219, thereby achieving a transformation efficiency of up to 1.25 × 10^6^ transformants/μg DNA. Using the optimized electroporation protocol, the gene editing plasmid pDX4383 was transferred into *P. elgii* 219, and the priming glycosyltransferase gene (*gene_4383*) was successfully inactivated using the group II intron plasmid pDX4383. Moreover, no bacteria adhered in the media. This work provided a systematic evaluation and improvement method for the electroporation-mediated transformation of exogenous DNA combined with a group II intron-based gene editing system that could serve as a workable program for the genetic engineering of *Paenibacillus* bacteria.

## Data Availability

*P. elgii* 219 has been stored in the China General Microbiological Culture Collection Center (preservation number CGMCC 32461), and the whole-genome information has been uploaded to the NCBI database (accession number PRJNA1193137).

## SUPPLEMENTAL MATERIAL

Supplemental material aem.02041-24-s0001.docx Tables S1 and S2; Figures S1 to S3.

ASM does not own the copyrights to Supplemental Material that may be linked to, or accessed through, an article. The authors have granted ASM a non-exclusive, world-wide license to publish the Supplemental Material files. Please contact the corresponding author directly for reuse.

## References

[1] Cochrane SA, Vederas JC. 2016. Lipopeptides from Bacillus and Paenibacillus spp.: a gold mine of antibiotic candidates. Med Res Rev 36:4–31. doi:10.1002/med.21321 24866700

[2] Daba GM, Elnahas MO, Elkhateeb WA. 2021. Contributions of exopolysaccharides from lactic acid bacteria as biotechnological tools in food, pharmaceutical, and medical applications. Int J Biol Macromol 173:79–89. doi:10.1016/j.ijbiomac.2021.01.110 33482209

[3] Baindara P, Chaudhry V, Mittal G, Liao LM, Matos CO, Khatri N, Franco OL, Patil PB, Korpole S. 2016. Characterization of the antimicrobial peptide penisin, a class Ia novel lantibiotic from Paenibacillus sp. strain A3. Antimicrob Agents Chemother 60:580–591. doi:10.1128/AAC.01813-15 26574006 PMC4704198

[4] Velkov T, Gallardo-Godoy A, Swarbrick JD, Blaskovich MAT, Elliott AG, Han M, Thompson PE, Roberts KD, Huang JX, Becker B, Butler MS, Lash LH, Henriques ST, Nation RL, Sivanesan S, Sani M-A, Separovic F, Mertens H, Bulach D, Seemann T, Owen J, Li J, Cooper MA. 2018. Structure, function, and biosynthetic origin of octapeptin antibiotics active against extensively drug-resistant Gram-negative bacteria. Cell Chem Biol 25:380–391. doi:10.1016/j.chembiol.2018.01.005 29396290 PMC6560181

[5] Liang TW, Wu CC, Cheng WT, Chen YC, Wang CL, Wang IL, Wang SL. 2014. Exopolysaccharides and antimicrobial biosurfactants produced by Paenibacillus macerans TKU029. Appl Biochem Biotechnol 172:933–950. doi:10.1007/s12010-013-0568-5 24122708 PMC3918387

[6] Schilling C, Klau LJ, Aachmann FL, Rühmann B, Schmid J, Sieber V. 2022. Structural elucidation of the fucose containing polysaccharide of Paenibacillus polymyxa DSM 365. Carbohydr Polym 278:118951. doi:10.1016/j.carbpol.2021.118951 34973768

[7] Aune TEV, Aachmann FL. 2010. Methodologies to increase the transformation efficiencies and the range of bacteria that can be transformed. Appl Microbiol Biotechnol 85:1301–1313. doi:10.1007/s00253-009-2349-1 19946685

[8] Ye S, Ma Z, Liu Z, Liu Y, Zhang M, Wang J. 2014. Effects of carbohydrate sources on biosorption properties of the novel exopolysaccharides produced by Arthrobacter ps-5. Carbohydr Polym 112:615–621. doi:10.1016/j.carbpol.2014.05.076 25129790

[9] Bach E, de Carvalho Fernandes G, Passaglia LMP. 2016. How to transform a recalcitrant Paenibacillus strain: from culture medium to restriction barrier. J Microbiol Methods 131:135–143. doi:10.1016/j.mimet.2016.10.012 27780731

[10] Rütering M, Cress BF, Schilling M, Rühmann B, Koffas MAG, Sieber V, Schmid J. 2017. Tailor-made exopolysaccharides-CRISPR-Cas9 mediated genome editing in Paenibacillus polymyxa. Synth Biol (Oxf) 2:ysx007. doi:10.1093/synbio/ysx007 32995508 PMC7445874

[11] Zhang H, Li Y, Chen X, Sheng H, An L. 2011. Optimization of electroporation conditions for Arthrobacter with plasmid PART2. J Microbiol Methods 84:114–120. doi:10.1016/j.mimet.2010.11.002 21078345

[12] Monk IR, Foster TJ. 2012. Genetic manipulation of Staphylococci-breaking through the barrier. Front Cell Infect Microbiol 2:49. doi:10.3389/fcimb.2012.00049 22919640 PMC3417578

[13] Haynes JA, Britz ML. 1990. The effect of growth conditions of Corynebacterium glutamicum on the transformation frequency obtained by electroporation. J Gen Microbiol 136:255–263. doi:10.1099/00221287-136-2-255

[14] Serafini F, Turroni F, Guglielmetti S, Gioiosa L, Foroni E, Sanghez V, Bartolomucci A, Motherway MO, Palanza P, van Sinderen D, Ventura M. 2012. An efficient and reproducible method for transformation of genetically recalcitrant bifidobacteria. FEMS Microbiol Lett 333:146–152. doi:10.1111/j.1574-6968.2012.02605.x 22640171

[15] Lu YP, Zhang C, Lv FX, Bie XM, Lu ZX. 2012. Study on the electro-transformation conditions of improving transformation efficiency for Bacillus subtilis. Lett Appl Microbiol 55:9–14. doi:10.1111/j.1472-765X.2012.03249.x 22486381

[16] Mougiakos I, Bosma EF, de Vos WM, van Kranenburg R, van der Oost J. 2016. Next generation prokaryotic engineering: the CRISPR-Cas toolkit. Trends Biotechnol 34:575–587. doi:10.1016/j.tibtech.2016.02.004 26944793

[17] Schilling C, Ciccone R, Sieber V, Schmid J. 2020. Engineering of the 2,3-butanediol pathway of Paenibacillus polymyxa DSM 365. Metab Eng 61:381–388. doi:10.1016/j.ymben.2020.07.009 32771627

[18] Meliawati M, Teckentrup C, Schmid J. 2022. CRISPR-Cas9-mediated large cluster deletion and multiplex genome editing in Paenibacillus polymyxa. ACS Synth Biol 11:77–84. doi:10.1021/acssynbio.1c00565 34914351

[19] Li O, Qian C-D, Zheng D-Q, Wang P-M, Liu Y, Jiang X-H, Wu X-C. 2015. Two UDP-glucuronic acid decarboxylases involved in the biosynthesis of a bacterial exopolysaccharide in Paenibacillus elgii. Appl Microbiol Biotechnol 99:3127–3139. doi:10.1007/s00253-014-6362-7 25573472

[20] Liu Y, Zheng H, Zhan G, Qin W, Tian L, Li W. 2014. Establishment of an efficient transformation protocol and its application in marine-derived Bacillus strain. Sci China Life Sci 57:627–635. doi:10.1007/s11427-014-4632-3 24771061

[21] Wen Z, Lu M, Ledesma-Amaro R, Li Q, Jin M, Yang S. 2020. Targetron technology applicable in solventogenic clostridia: revisiting 12 years' advances. Biotechnol J 15:e1900284. doi:10.1002/biot.201900284 31475782

[22] Frazier CL, San Filippo J, Lambowitz AM, Mills DA. 2003. Genetic manipulation of Lactococcus lactis by using targeted group II introns: generation of stable insertions without selection. Appl Environ Microbiol 69:1121–1128. doi:10.1128/AEM.69.2.1121-1128.2003 12571038 PMC143682

[23] Yao J, Lambowitz AM. 2007. Gene targeting in gram-negative bacteria by use of a mobile group II intron (“Targetron”) expressed from a broad-host-range vector. Appl Environ Microbiol 73:2735–2743. doi:10.1128/AEM.02829-06 17322321 PMC1855620

[24] Jia K, Zhu Y, Zhang Y, Li Y. 2011. Group II intron-anchored gene deletion in Clostridium. PLoS ONE 6:e16693. doi:10.1371/journal.pone.0016693 21304965 PMC3031624

[25] Atrazhev AM, Elliott JF. 1996. Simplified desalting of ligation reactions immediately prior to electroporation into E. coli. BioTechniques 21:1024. doi:10.2144/96216bm12 8969827

[26] Novotny R, Berger H, Schinko T, Messner P, Schäffer C, Strauss J. 2008. A temperature-sensitive expression system based on the Geobacillus stearothermophilus NRS 2004/3a sgsE surface-layer gene promoter. Biotechnol Appl Biochem 49:35–40. doi:10.1042/BA20070083 17576197 PMC4389859

[27] Dunny GM, Lee LN, LeBlanc DJ. 1991. Improved electroporation and cloning vector system for gram-positive bacteria. Appl Environ Microbiol 57:1194–1201. doi:10.1128/aem.57.4.1194-1201.1991 1905518 PMC182867

[28] Zhang Z, Ding ZT, Shu D, Luo D, Tan H. 2015. Development of an efficient electroporation method for iturin A-producing Bacillus subtilis ZK. Int J Mol Sci 16:7334–7351. doi:10.3390/ijms16047334 25837631 PMC4425020

[29] Yi Y, Kuipers OP. 2017. Development of an efficient electroporation method for rhizobacterial Bacillus mycoides strains. J Microbiol Methods 133:82–86. doi:10.1016/j.mimet.2016.12.022 28042055

[30] Tryfona T, Bustard MT. 2006. Enhancement of biomolecule transport by electroporation: a review of theory and practical application to transformation of Corynebacterium glutamicum. Biotechnol Bioeng 93:413–423. doi:10.1002/bit.20725 16224791

[31] Turgeon N, Laflamme C, Ho J, Duchaine C. 2006. Elaboration of an electroporation protocol for Bacillus cereus ATCC 14579. J Microbiol Methods 67:543–548. doi:10.1016/j.mimet.2006.05.005 16820234

[32] Peng D, Luo Y, Guo S, Zeng H, Ju S, Yu Z, Sun M. 2009. Elaboration of an electroporation protocol for large plasmids and wild-type strains of Bacillus thuringiensis. J Appl Microbiol 106:1849–1858. doi:10.1111/j.1365-2672.2009.04151.x 19291242

[33] Vojcic L, Despotovic D, Martinez R, Maurer KH, Schwaneberg U. 2012. An efficient transformation method for Bacillus subtilis DB104. Appl Microbiol Biotechnol 94:487–493. doi:10.1007/s00253-012-3987-2 22395911

[34] Zhang G, Bao P, Zhang Y, Deng A, Chen N, Wen T. 2011. Enhancing electro-transformation competency of recalcitrant Bacillus amyloliquefaciens by combining cell-wall weakening and cell-membrane fluidity disturbing. Anal Biochem 409:130–137. doi:10.1016/j.ab.2010.10.013 20951110

[35] Huang H, Liu Z, Qiu Y, Wang X, Wang H, Xiao H, Lu Z. 2021. Efficient electrotransformation of Rhodococcus ruber YYL with abundant extracellular polymeric substances via a cell wall-weakening strategy. FEMS Microbiol Lett 368:fnab049. doi:10.1093/femsle/fnab049 33974050

[36] Pyne ME, Moo-Young M, Chung DA, Chou CP. 2013. Development of an electrotransformation protocol for genetic manipulation of Clostridium pasteurianum. Biotechnol Biofuels 6:50. doi:10.1186/1754-6834-6-50 23570573 PMC3658993

[37] Hammes W, Schleifer KH, Kandler O. 1973. Mode of action of glycine on the biosynthesis of peptidoglycan. J Bacteriol 116:1029–1053. doi:10.1128/jb.116.2.1029-1053.1973 4200845 PMC285483

[38] Bonnassie S, Burini JF, Oreglia J, Trautwetter A, Patte JC, Sicard AM. 1990. Transfer of plasmid DNA to Brevibacterium lactofermentum by electrotransformation. J Gen Microbiol 136:2107–2112. doi:10.1099/00221287-136-10-2107 2269876

[39] Ye X, Dong H, Huang YP. 2014. Highly efficient transformation of Stenotrophomonas maltophilia S21, an environmental isolate from soil, by electroporation. J Microbiol Methods 107:92–97. doi:10.1016/j.mimet.2014.09.010 25300664

[40] Wang C, Cui Y, Qu X. 2020. Optimization of electrotransformation (ETF) conditions in lactic acid bacteria (LAB). J Microbiol Methods 174:105944. doi:10.1016/j.mimet.2020.105944 32417130

[41] Murray KD, Aronstein KA. 2008. Transformation of the Gram-positive honey bee pathogen, Paenibacillus larvae, by electroporation. J Microbiol Methods 75:325–328. doi:10.1016/j.mimet.2008.07.007 18687369

