# Supplementary material for: Construction of an efficient electroporation transformation system promotes the application of Targetron in wild-type Paenibacillus elgii 219
Source: Appl Environ Microbiol. 2025 Apr 22;91(5):e02041-24. doi: 10.1128/aem.02041-24 (PMC12093971; doi:10.1128/aem.02041-24)
Supplement: Supplemental material — Tables S1 and S2; Figures S1 to S3. [file aem.02041-24-s0001.docx]

**Supplementary Materials**

**The construction of an efficient electroporation transformation system promotes the application of Targetron in wild-type *Paenibacillus elgii* 219**

Guangxin Yang^1,2^, Siyu Li^1,2^, Yonghang Ma^1,2^, Siyi Peng^1,2^, Xiangfang Zeng^1,2^, Jinxiu Huang^3,4^, AiHua Deng^1,2^, Shiyan Qiao^1,2^, and Haitao Yu^1,2,^*

^1^State Key Laboratory of Animal Nutrition and Feeding, Ministry of Agriculture and Rural Affairs Feed Industry Centre, China Agricultural University, Beijing 100193, PR. China.

^2^ Frontier Technology Research Institute of China Agricultural University in Shenzhen, Shenzhen, 518119, China;

^3^ Chongqing Academy of Animal Science, Rongchang, Chongqing 40240, China;

^4^ National Center of Technology Innovation for Pigs, Rongchang, Chongqing 40240, China.

* Corresponding author: Haitao Yu (Yu H. T.).

E-mail: yuhaitao@cau.edu.cn (H.Y)

**Table S1. Primers and gBlock sequence.**

| Name | Sequence (5`-3`) | Description |
| --- | --- | --- |
| P1 | cataaacacaagtgataatcatggtcatagctgtttcctg | Linearized vector pWAe |
| P2 | atatgcctgtaacagatctagatgaacgtttcaagccttg |  |
| P3 | ctgttacaggcatattcatatcaatg | Amplified group II intron box |
| P4 | tcacttgtgtttatgaatcacgtga |  |
| P5 | cgcggaccgataaagctcatgtc | Verified inactivate mutants |
| P6 | ctaccgtggaatacgcgctaag |  |
| **gBlock for group II intron (including PsgsE)** | | |
| ctgttacaggcatattcatatcaatgtcgccatcgatggaaataatttcaatggcatattctctaaaaaatttgtatttagggctagaatttagaaagaaaatgtattattatagaagacgtagaacattaaggaatgatgtgaaagtgctagcactgaatgatagccttgctaataagtctatcatctggacacacaggatgatcttggttcatgttagcaccttttaggcaatgaatctactaaacggcagccgtttaggatcattgtcttaaagtttcataaaaacgaaagcttataattatccttagatttcgagcatgtgcgcccagatagggtgttaagtcaagtagtttaaggtactactctgtaagataacacagaaaacagccaacctaaccgaaaagcgaaagctgatacgggaacagagcacggttggaaagcgatgagttacctaaagacaatcgggtacgactgagtcgcaatgttaatcagatataaggtataagttgtgtttactgaacgcaagtttctaatttcgattaaatctcgatagaggaaagtgtctgaaacctctagtacaaagaaaggtaagttacaatgctcgacttatctgttatcaccacatttgtacaatctgtaggagaacctatgggaacgaaacgaaagcgatgccgagaatctgaatttaccaagacttaacactaactggggataccctaaacaagaatgcctaatagaaaggaggaaaaaggctatagcactagagcttgaaaatcttgcaagggtacggagtactcgtagtagtctgagaagggtaacgccctttacatggcaaaggggtacagttattgtgtactaaaattaaaaattgattagggaggaaaacctcaaaatgaaaccaacaatggcaattttagaaagaatcagtaaaaattcacaagaaaatatagacgaagtttttacaagactttatcgttatcttttacgtccagatatttattacgtggcgacgcgttgggaaatggcaatgatagcgaaacaacgtaaaactcttgttgtatgctttcattgtcatcgtcacgtgattcataaacacaagtgaatgtcgacagtgaatttttacgaacgaacaataacagagccgtatactccgagaggggtacgtacggttcccgaagagggtggtgcaaaccagtcacagtaatgtgaacaaggcggtacctccctacttcaccatatcattttctgcagccccctagaaataattttgtttaactttaagaaggagatatacatatatggctagatcgtccattccgacagcatcgccagtcactatggcgtgctgctagcgctatatgcgttgatgcaatttctatgcactcgtagtagtctgagaagggtaacgccctttacatggcaaaggggtacagttattgtgtactaaaattaaaaattgattagggaggaaaacctcaaaatgaaaccaacaatggcaattttagaaagaatcagtaaaaattcacaagaaaatatagacgaagtttttacaagactttatcgttatcttttacgtccagatatttattacgtggcgtatcaaaatttatattccaataaaggagcttccacaaaaggaatattagatgatacagcggatggctttagtgaagaaaaaataaaaaagattattcaatctttaaaagacggaacttactatcctcaacctgtacgaagaatgtatattgcaaaaaagaattctaaaaagatgagacctttaggaattccaactttcacagataaattgatccaagaagctgtgagaataattcttgaatctatctatgaaccggtattcgaagatgtgtctcacggttttagacctcaacgaagctgtcacacagctttgaaaacaatcaaaagagagtttggcggcgcaagatggtttgtggagggagatataaaaggctgcttcgataatatagaccacgttacactcattggactcatcaatcttaaaatcaaagatatgaaaatgagccaattgatttataaatttctaaaagcaggttatctggaaaactggcagtatcacaaaacttacagcggaacacctcaaggtggaattctatctcctcttttggccaacatctatcttcatgaattggataagtttgttttacaactcaaaatgaagtttgaccgagaaagtccagaaagaataacacctgaatatcgggagctccacaatgagataaaaagaatttctcaccgtctcaagaagttggagggtgaagaaaaagctaaagttcttttagaatatcaagaaaaacgtaaaagattacccacactcccctgtacctcacagacaaataaagtattgaaatacgtccggtatgcggacgacttcattatctctgttaaaggaagcaaagaggactgtcaatggataaaagaacaattaaaactttttattcataacaagctaaaaatggaattgagtgaagaaaaaacactcatcacacatagcagtcaacccgctcgttttctgggatatgatatacgagtaaggagatctggaacgataaaacgatctggtaaagtcaaaaagagaacactcaatgggagtgtagaactccttattcctcttcaagacaaaattcgtcaatttatttttgacaagaaaatagctatccaaaagaaagatagctcatggtttccagttcacaggaaatatcttattcgttcaacagacttagaaatcatcacaatttataattctgaactccgcgggatttgtaattactacggtctagcaagtaattttaaccagctcaattattttgcttatcttatggaatacagctgtctaaaaacgatagcctccaaacataagggaacactttcaaaaaccatttccatgtttaaagatggaagtggttcgtgggggatcccgtatgagataaagcaaggtaagcagcgccgttattttgcaaattttagtgaatgtaaatccccttatcaatttacggatgagataagtcaagctcctgtattgtatggctatgcccggaatactcttgaaaacaggttaaaagctaaatgttgtgaattatgtgggacgtctgatgaaaatacttcctatgaaattcaccatgtcaataaggtcaaaaatcttaaaggcaaagaaaaatgggaaatggcaatgatagcgaaacaacgtaaaactcttgttgtatgctttcattgtcatcgtcacgtgattcataaacacaagtga | | |

**Table S2. Results of guided glycosyltransferase BlastP comparison**

| Gene Name | Accession | Gene No. | Identity  (%) | Coverage  (%) | E-value | Score |
| --- | --- | --- | --- | --- | --- | --- |
| *GTp* | WP_054974698 | 4383 | 99 | 99.5 | 7.47E-152 | 1084 |

Note: The deduced proteins were analyzed with a BLASTP homology search, and *gene_4383* was mapped to the genome of *P. elgii* 219 to be 99% similar to a guide glycosyltransferase (*GTp*, accession WP_054974698) from *Paenibacillus sp*. A3**.**


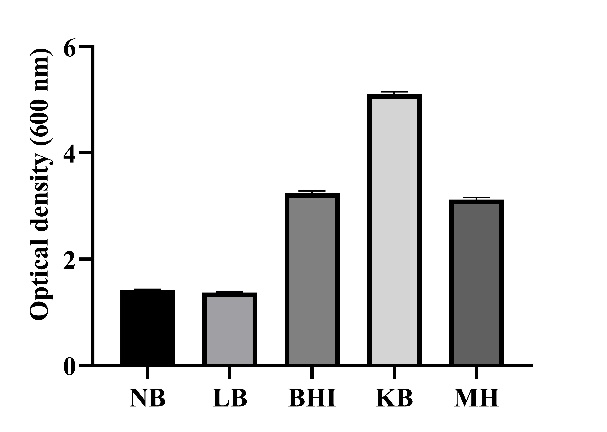


**Figure S1. Maximum OD_600_ value of *P. elgii* 219 in medium.** *P. elgii* 219 was activated in NB medium overnight, and the next day was transferred to a shaker containing 50mL medium at 4% inoculated volume and cultured at 37℃ at 220 rpm until plateau stage. All experiments were independently repeated three times. NB: nutrient broth medium; LB: Luria-Bertani; BHI: Brian Heart Infusion; KB: King broth medium; MH: Müller-Hinton medium.


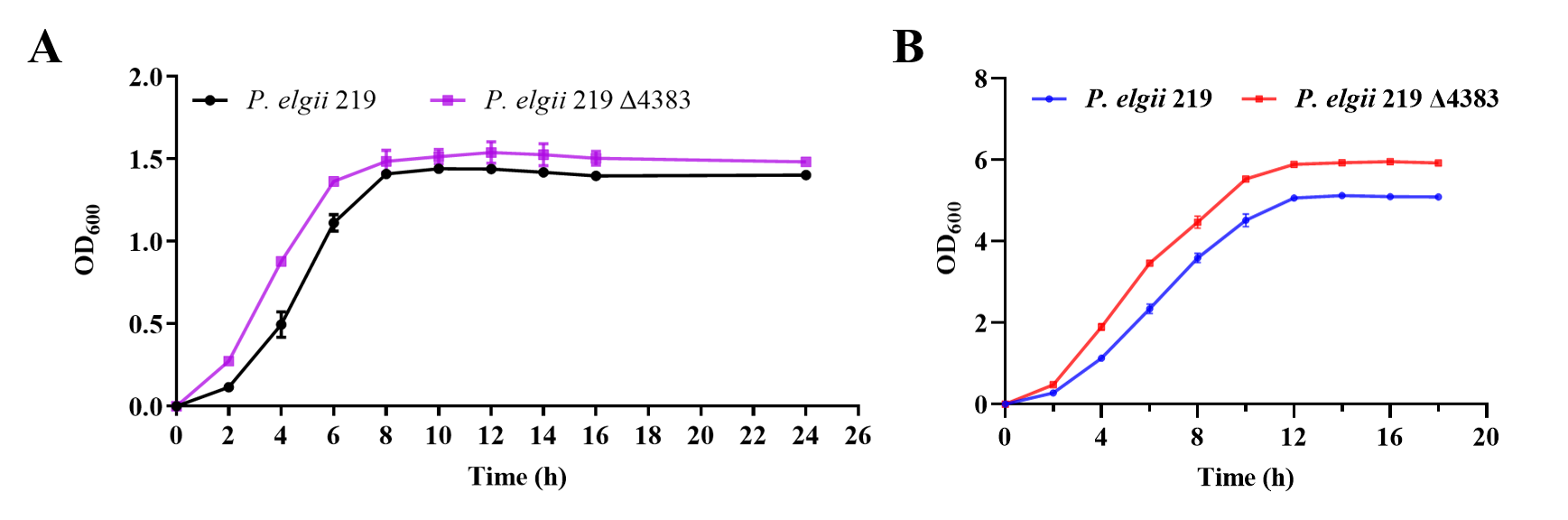


**Figure S2. Growth rate of *P. elgii* 219 Δ4383.** *P. elgii* 219 and *P. elgii* 219 Δ4383 cultured in NB and KB medium with 4% (v/v) inoculation volume, OD600 values were recorded per 1 h.


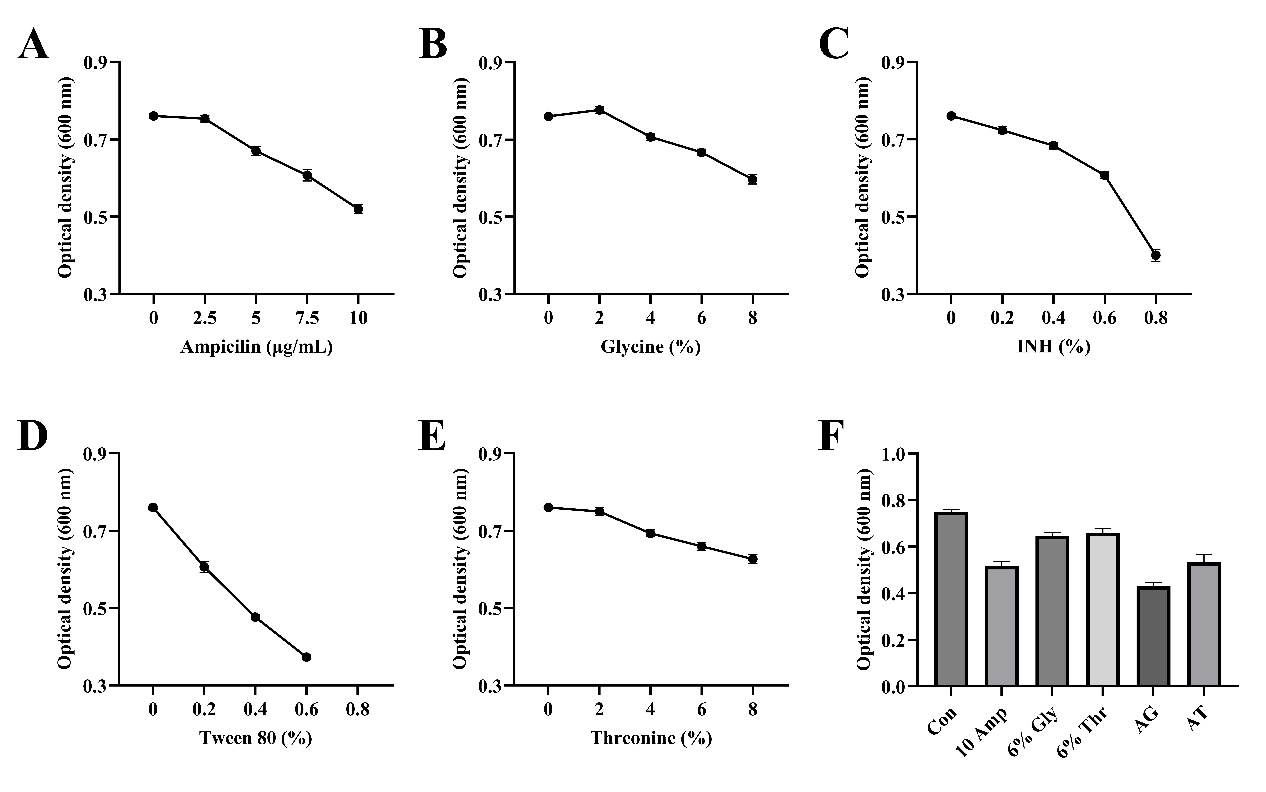


**Figure S3. Effect of cell wall attenuator treatment on growth of *P. elgii* 219.** *P. elgii* 219 was grown in KB medium to OD_600_ = 0.5, then cell-wall weakening agents were added and cultured for another 1 h to record OD_600_ values.
